# Automatic quantification of lymphocyte vacuolization in peripheral blood smears of patients with Batten's disease (CLN3 disease)

**DOI:** 10.1002/jmd2.12191

**Published:** 2021-01-25

**Authors:** Lourens J. P. Nonkes, Willemijn F. E. Kuper, Karin Berrens‐Hogenbirk, Ruben E. A. Musson, Peter M. van Hasselt, Albert Huisman

**Affiliations:** ^1^ Central Diagnostic Laboratory University Medical Center Utrecht Utrecht The Netherlands; ^2^ Department of Metabolic Diseases, Wilhelmina Children's Hospital, University Medical Center Utrecht Utrecht University Utrecht The Netherlands

**Keywords:** CLN3 disease, lymphocyte vacuolization, machine learning, neuronal ceroid lipofuscinosis

## Abstract

Quantifying lymphocyte vacuolization in peripheral blood smears (PBSs) serves as a measure for disease severity in CLN3 disease—a lysosomal storage disorder of childhood‐onset. However, thus far quantification methods are based on labor‐intensive manual assessment of PBSs. As machine learning techniques like convolutional neural networks (CNNs) have been deployed quite successfully in detecting pathological features in PBSs, we explored whether these techniques could be utilized to automate quantification of lymphocyte vacuolization. Here, we present and validate a deep learning pipeline that automates quantification of lymphocyte vacuolization. By using two CNNs in succession, trained for cytoplasm‐segmentation and vacuolization‐detection, respectively, we obtained an excellent correlation with manual quantification of lymphocyte vacuolization (*r* = 0.98, n = 40). These results show that CNNs can be utilized to automate the otherwise cumbersome task of manually quantifying lymphocyte vacuolization, thereby aiding prompt clinical decisions in relation to CLN3 disease, and potentially beyond.

**Synopsis**

A deep learning pipeline is presented that can be utilized to automate lymphocyte vacuolization, a pathological hallmark in CLN3 disease that serves as a measure for disease severity.

## 1 INTRODUCTION

CLN3 disease (OMIM #204200) is a lysosomal disorder of childhood onset. Although relatively rare, it represents a major cause of childhood dementia.^1^ One of the hallmarks of CLN3 disease is the presence of lymphocyte vacuolization in peripheral blood smears (PBSs). We have recently shown that the extent of vacuolization is related to disease severity.^2^ Thus, although the degree of vacuolization does not seem to relate to disease progression in individual patients, there is a relationship with disease severity in a sense that in general patients with a severe phenotypic variant (ie, classical CLN3) show increased vacuolization compared to patients expressing a milder disease variant (ie, protracted CLN3), or healthy controls that express low levels of vacuolization. As such, we perform manual quantifications of lymphocyte vacuolization in PBSs of patients suspected of CLN3 disease to aid diagnosis. Although manual quantification yields reasonable results, we observed that it can be notoriously difficult to exclude subjectivity altogether from the analysis. To circumvent this issue, we request at least two technicians to assess the same blood smear for lymphocyte vacuolization, averaging the results, making the endeavor quite time consuming. Hence, the possibility to automate these analyses in an objective manner would be a valuable asset for the diagnostic laboratory. Machine learning methods like convolutional neural networks (CNNs) have proven to be quite successful in detecting image features and could potentially be utilized to automate (aspects of) PB analyses. Indeed, CNNs have been used to differentiate between leukocytes,^3^ and to detect myelodysplastic syndromes in PBSs.^4^ In this study, we present an automated pipeline based on CNNs that enabled us to successfully detect and quantify lymphocyte vacuolization as observed in PBSs of CLN3 disease patients.

## 2 METHODS

Images of May‐Grünwald‐Giemsa stained lymphocytes in PBSs were acquired using a Cellavision DM1200 digital microscope (Cellavision AB, Lund, Sweden) and subsequently fed into the deep learning pipeline composed of two subsequent CNNs. The first CNN (segmentation CNN [sCNN]), with an U‐Net architecture,^5^ was trained to identify cellular and nuclear boundaries so that a cytoplasm segmentation mask could be made, that is, blacking out all of the image except the cytoplasm of the lymphocyte of interest. Subsequently, segmented images were fed to a second CNN (quantification CNN [qCNN]), based on a Resnet‐50 architecture,^6^ trained to identify and quantify lymphocyte vacuolization. This segmentation‐before‐quantification approach was used as preliminary findings had shown that cytoplasm segmentation prior to analysis improved qCNN performance, likely by removing irrelevant features from the image. In order to train the sCNN to construct cell segmentation masks, a total of 373 lymphocytes and 28 neutrophilic granulocytes were randomly selected and manually segmented using ImageJ v1.51.^7^ To improve performance all these images were augmented 20 times, resulting in a set of 8020 example images for supervised training of the CNN. Image augmentation itself was achieved by rotation, shifting, shearing, zooming, and flipping the original images. For nucleus mask formation, sCNN training was based on 271 and 81 randomly selected and manually segmented images of lymphocytes and neutrophilic granulocytes, respectively. Data sets for cell and nucleus segmentation training/validation partially overlapped (n = 66) and were based on a 80/20% training/validation split. sCNN model performance was evaluated using the dice coefficient (dice coefficient = 2 * the area of overlap between manually and sCNN generated mask, divided by the total number of pixels in both images). This statistic can be used to measure the similarity between the binary manually drawn and sCNN generated mask, 0 indicating no spatial overlap and 1 complete overlap. Acceptable dice coefficients of 0.96 and 0.95 were obtained for the nucleus‐ and cell‐mask sCNN model on their respective validation sets. A cytoplasm segmentation mask was constructed by subtracting the nucleus mask from the cell segmentation mask. The qCNN had an modified ResNet‐50 architecture, in which the original 1000‐neuron dense layer on top of the convolutional base was replaced by a 2‐neuron softmax activation layer which gave the output of the CNN as a probability vector (ie, probability of cell being a vacuolated lymphocyte). The qCNN was subsequently trained to discriminate between healthy and vacuolated lymphocytes, using 983 selected (segmented) images of vacuolated lymphocytes from CLN3 disease patients (n = 10) and 5684 (segmented) images of randomly chosen lymphocytes from healthy controls (n = 32). Vacuolated patient lymphocytes on which two experienced experts agreed were included in the dataset. Vacuolization was defined as the presence of evident round clearings in the cytoplasm of the lymphocytes. A 80/20% training/validation split was used and a 96% accuracy on the validation set was obtained after 21 epochs of training. Additional epochs did not improve accuracy. All algorithms were written in Python (Spyder v3.3.2, Python v3.6) utilizing Keras and Tensorflow libraries for neural net deployment. After training both CNNs, we quantified 40 PBSs of CLN3 disease patients (n = 36) and healthy controls (n = 4) using the automated pipeline and compared outcomes with that of manual quantification, the latter being the averaged result of two separate analyses on the same images, performed by two experienced experts. The data set used for the method comparison was based on a separate set of blood smears and thus entirely independent of the initial training/validation set. There was however a partial overlap in that separate blood smears from the same patient were in both the initial training/validation set and this subsequent validation set (n = 5). This practical limitation is due to the limited number of available Batten patients. For the qCNN, a probability level of ≥65% was considered specific for vacuolization. Results were evaluated using AnalyseIt (v4.80.9) by means of a Passing‐Bablok regression analysis and Bland Altman plot. Confidence interval (CI) of Passing‐Bablok analysis was based on 999 bootstrap samples. To investigate whether method variances were comparable, differences between paired measurements were analyzed using a Levene's test (neural net% − manual 1% vs neural net% − manual 2% vs manual 1% − manual 2%).

## 3 RESULTS

**FIGURE 1:**
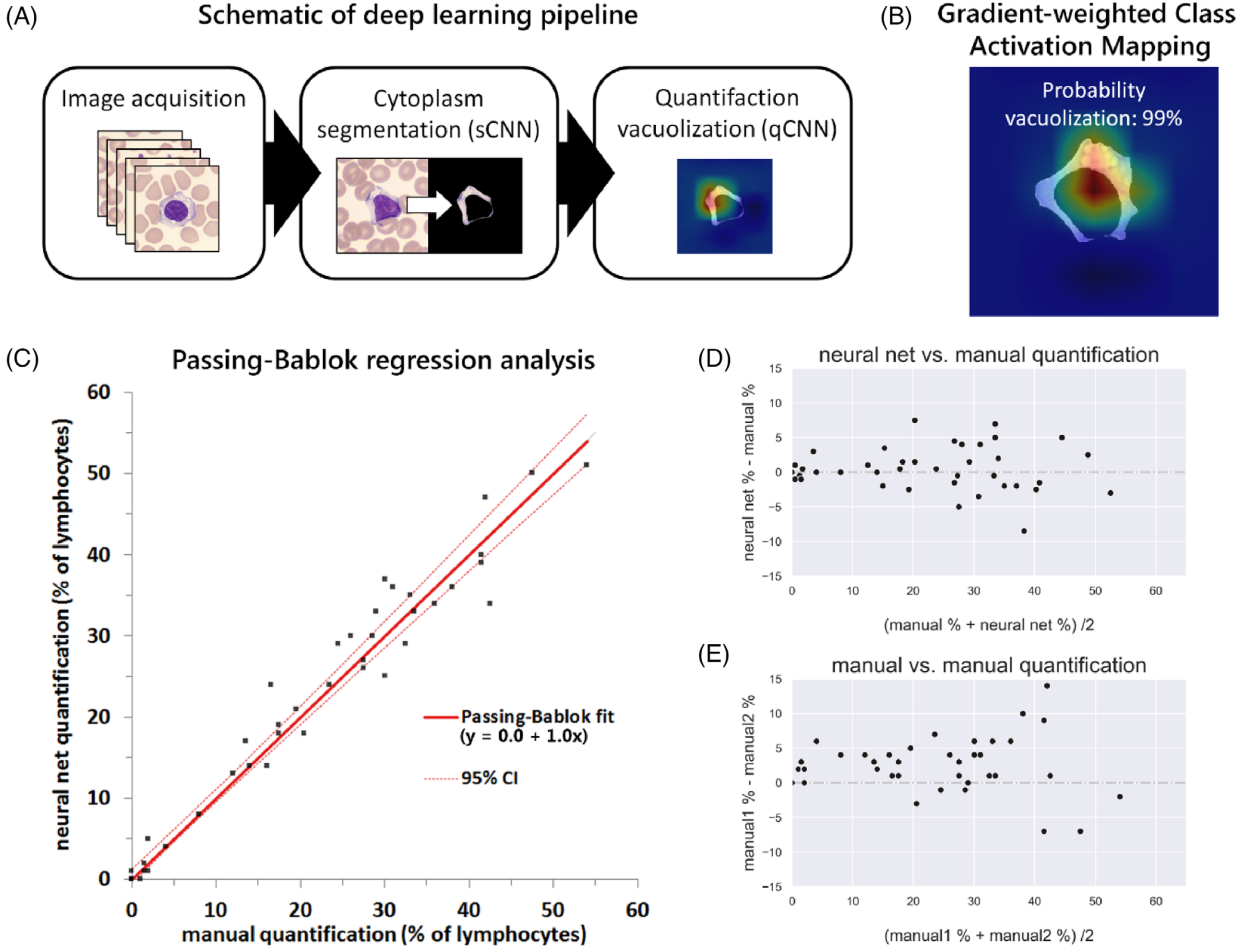
A, Schematic illustration of the deployed deep learning pipeline. B, Representative example Gradient‐weighted Class Activation Mapping (Grad‐CAM) image for utilized qCNN, combined with reported probability for vacuolization. C, Passing‐Bablok analysis and, D, Bland Altman plot for comparison neural net vs manual quantification, the latter being the average of two separate analyses. E, Bland Altman plot for the separate manual analyses. qCNN, quantification convolutional neural network

Figure 1A shows a schematic overview of the utilized deep learning pipeline in this study. Gradient‐weighted Class Activation Mapping (Grad‐CAM), a technique to visualize input regions “important” and utilized by the CNN for predictions,^8^ identified vacuolated regions in the cytoplasm of lymphocytes as important for the qCNN to make predictions (Figure 1B).

Thus, the locations of the highest gradient appear to coincide with the image regions containing vacuoles. Comparison between CNN predictions and manual results showed an excellent correlation between the two methods (Pearson *r* = 0.98; Figure 1C). The slope coefficient of the Passing‐Bablok fit was equal to 1.0 (95% CI = 0.9‐1.1), indicating no proportional difference between the two methods. In addition, the intercept was 0.0 (95% CI = −0.5 to 1.3) indicating no significant bias between methods. The observed variance between our novel method and the current manual method was not substantially different from that observed between the two manual quantifications (Levene's test *P* = .51; see also Figure 1D,E).

## 4 DISCUSSION AND CONCLUSIONS

Here, we demonstrate a fully automated approach to quantify lymphocyte vacuolization as observed in PBSs of CLN3 disease patients. The availability of such an automated method in the lab would speed up the analysis and reporting back to clinic, aiding timely diagnosis. Of interest, lymphocyte vacuolization has also been observed in other metabolic disorders like sialidosis type 2 and α‐mannosidosis.^9^ The ability to automatically screen and quantify vacuolization in these and other disorders associated with lymphocyte vacuolization may open up new venues for research. Indeed, preliminary results from our lab suggest that, for example, α‐mannosidosis associated vacuolization can be readily quantified using our automated method. However, the model should be properly validated before being used on blood smears of patients with other disorders associated with vacuolization. The same holds for quantifying the degree of vacuolization in alternative cell types like monocytes. As such, our model is currently validated only for quantifying vacuolization in lymphocytes of CLN3 patients. Nevertheless, if enough training examples are available, it is relatively easy to retrain the qCNN for alternative cell types and/or pathophysiological cellular features. As such, we believe that our automated pipeline can be considered a useful and versatile technological addendum to aid clinical decisions in relation to CLN3 disease, and beyond.

## CONFLICT OF INTEREST

The authors declare no potential conflict of interest.

## ETHICS STATEMENT

This article does not contain any studies with human or animal subjects performed by any of the authors.

## Data Availability

Upon publication, code and models will be shared publically on Github (https://github.com/Nonkes).
